# NRJ Media as the Gold-Standard *Arcobacter*-Specific Detection System: Applications in Poultry Testing

**DOI:** 10.3389/fmicb.2022.903079

**Published:** 2022-06-21

**Authors:** Paul T. Nguyen, Karina Tuz, Lawrence Restaino, Oscar Juárez

**Affiliations:** ^1^R & F Products, Inc., Downers Grove, IL, United States; ^2^Department of Biology, Illinois Institute of Technology, Chicago, IL, United States

**Keywords:** *Arcobacter*, selective media, 16S amplicon sequencing, poultry, microbiome, culture-based detection

## Abstract

*Arcobacter* species are ubiquitous emerging pathogens with an impact that has been underestimated due to limitations in isolation and detection methods. Our group recently developed the novel NRJ *Arcobacter*-detection system, with major improvements in specificity and selectivity compared to other culture-based methods. In this work, the NRJ detection system was evaluated using retail whole broiler chicken carcass. Nanopore 16S rRNA gene amplicon sequencing demonstrated that *Arcobacter* species are found in very low abundance in retail chicken and that indigenous microbiota could be a major factor interfering with detection. Comparison of the microbiome obtained from modified Houf broth (HB) method, as the standard detection system, and the novel NRJ method, showed *Arcobacter* abundances of <15% and >97%, respectively. The NRJ system significantly inhibits the growth of non-target microbiota, and specifically allows the multiplication of *Arcobacter* species. In this report, we describe the gold-standard of *Arcobacter*-specific culture-based method to test food matrices, which can be used for other applications, such as clinical and environmental sampling.

## Introduction

*Arcobacter* species are emerging foodborne pathogens that cause bacterial gastroenteritis and bacteremia in severe cases (Collado and Figueras, 2011; Chieffi et al., 2020). These microorganisms have been identified as the causal agent of foodborne illnesses (Hsueh et al., 1997; Wybo et al., 2004; Arguello et al., 2015) and outbreaks worldwide (Vandamme et al., 1992; Lappi et al., 2013). In a 5-year period prevalence study, *Arcobacter* spp. was found to be the fourth most prevalent gastrointestinal pathogen (1.3%) after *Campylobacter* (5.6%), *Salmonella* spp. (2.0%), and *Clostridum difficile* (1.6%; Van den Abeele et al., 2014). Although the pathogenicity mechanisms employed by these microorganisms are largely unknown, infections caused by *Arcobacter* species display similar clinical features to the closely-related and well-established pathogenic *Campylobacter* species (Vandenberg et al., 2004; On et al., 2020). In fact, *Campylobacter*-putative virulence determinants for invasion, adhesion, and cytotoxicity have been identified in *Arcobacter* (Girbau et al., 2015; Šilha et al., 2015). Consumption of contaminated chicken meat is a common route of transmission of *Arcobacter* in humans (Ramees et al., 2017), in part due to a highly variable prevalence in retail chicken, ranging from 10.4% to 56.0% worldwide (Barboza et al., 2017; Jribi et al., 2020). Although various factors influence *Arcobacter* identification (e.g., geographical location, condition of the sample, and sampling procedures), the lack of a standard and highly specific isolation and detection methods are major issues that obscure *Arcobacter* prevalence, which leads to the underestimation of its impact to human health (Prouzet-Mauléon et al., 2006; Snelling et al., 2006).

Due to its importance, several detection systems have been developed to isolate *Arcobacter* spp. from food matrices, including those described by Collins et al. (1996), DeBoer et al. (1996), Atabay and Corry (1998), Johnson and Murano (1999), and Houf et al. (2001). The efficacy of these methods were evaluated in various comparison studies, and it was determined that the modified Houf Broth (HB) method, comprised of the selective Houf enrichment broth and modified charcoal cefoperazone deoxycholate agar supplemented with three antibiotics, was the most sensitive and specific among *Arcobacter* culture-based methods (Ohlendorf and Murano, 2002; Hamill et al., 2008; Merga et al., 2011). However, this method shows a high prevalence of non-target antibiotic-resistant microbiota as common contaminants (Merga et al., 2011), which compromises recovery and detection, and hinders the use of modified HB as a standard method. The novel chromogenic Nguyen-Restaino-Juárez (NRJ) detection system was proposed by our research group, as an *Arcobacter-selective* media that inhibits the growth of various microorganisms found in food (Nguyen et al., 2020). NRJ media increases the efficacy of isolating *Arcobacter* spp. from food products through the use of selective antibiotics in the culture media and the incorporation of a chromogenic substrate in the plating agar that targets the presence of C-2 esterase activity. The combination of the selective and differential agents reduces growth of contaminants that interferes with colony isolation and allows for easy detection of presumptive *Arcobacter* colonies in the plating medium. Nevertheless, the NRJ detection system has not been evaluated for standard laboratory or commercial use. In this work, we used 16S rRNA gene amplicon sequencing to compare the efficiency of NRJ and modified HB media to detect and isolate *Arcobacter* colonies in retail poultry. Our results show that the relative abundance of *Arcobacter* in modified HB is <15%, which is inadequate for reliable detection. On the other hand, *Arcobacter* abundance in NRJ plates was >97%. In this work, we show that NRJ vastly outperforms modified HB, and within the limits of the study, it can be considered the first *Arcobacter-*specific detection and isolation system reported. The novel NRJ detection system could be used in various food, clinical and environmental applications to ascertain *Arcobacter* occurrence, prevalence and significance as a human pathogen.

## Materials and Methods

### Sample Collection and Analysis

Samples of whole broiler chicken carcasses (2.4-kg) were purchased at local markets in Dupage County, IL. A total of 30 samples were analyzed within 24 h upon receipt. Preparation of individual carcass samples was performed with initial removal of the giblet pouch from the chicken body cavity. Whole broiler chicken carcasses were aseptically placed in sterile stomacher bags with 400 ml of Buffered Peptone Water (BPW, BD Difco Laboratories, Detroit, MI) and hand-massaged for 2 min to ensure the whole surface of the samples were exposed to the rinse. Aliquots (100 ml) of collected chicken rinsate were centrifuged at 10,000 × g for 8 min to pellet bacterial cells. Recovered cells were washed twice with 0.85% saline solution and resuspended in 5 ml. Resuspended cells were pooled into a composite sample consisting of 10 individual whole broiler chicken carcasses. Pooled samples were stored at 2°C–8°C and bacterial DNA was extracted within 24 h. Microbial content of each sample was assessed by mesophilic aerobic plate count using Plate Count Agar (PCA, Neogen, Lansing, MI, United States), which was prepared according to the manufacturer’s instruction. A 0.1 ml aliquot of chicken rinsate was spread-plated on PCA at the serial dilutions of 10^−1^ to 10^−3^ using 0.85% saline. Plates were incubated aerobically at 35°C for 48 h (Salfinger and Tortorello, 2015). Bacterial enumeration for each sample was carried out in triplicate. Bacterial enumeration of each individual chicken sample was log-transformed and the mean of the pooled samples was reported as the mean ± standard deviation (Gao and Martos, 2018).

### Culture Media Preparation

Modified HB was prepared as described by Merga et al., (2011). NRJ media were prepared according to the methods reported previously by our group (Nguyen et al., 2020). NRJ broth was prepared with a proprietary basal broth medium (R&F Products, Inc., Downers Grove, IL), autoclaved at 121°C for 15 min, and allowed to cool to room temperature. The NRJ-*Arcobacter* chromogenic plating medium (R&F Products, Inc., Downers Grove, IL), was prepared with a proprietary basal agar medium and heated to a full boil. The medium was cooled to 50°C and supplemented with the Aldol acetate chromogen substrate (Biosynth AG, Staad, Switzerland, 70 μg/ml) that targets C-2 esterase. Selective agents were aseptically added to NRJ media according methods reported by our group (Nguyen et al., 2020) with one minor modification. Moxalactam (Research Products International, Mount Prospect, IL) concentration in the single strength (1X) NRJ broth was increased to 64 mg/l, which was necessary to inhibit the growth of non-target microorganisms. All prepared agar media were poured into Petri plates, dried at room temperature under dark conditions for 24–48 h, and stored at 2°C–8°C for up to 14 days. Plates were allowed to acclimate at room temperature for at least 15 min prior to use.

### Detection of *Arcobacter* spp. From Chicken Carcass

Direct enumeration was performed to evaluate the isolation of *Arcobacter* spp. from chicken rinsate samples using selective agar plates of modified HB and NRJ. Aliquots (0.1 ml) of chicken rinsate were spread-plated onto modified HB and NRJ agar plates and incubated aerobically at 30°C for 48 h (modified HB) and 72 h (NRJ), as described previously (Merga et al., 2011; Nguyen et al., 2020). After incubation, 1.0 ml of 0.85% saline solution was dispensed on the surface of each plate and a sterile L-shaped glass spreader was used to dislodge the bacterial lawn from the plating medium. 1.0-ml aliquots containing the bacterial lawn on the plating media were aseptically recovered and bacterial cells from the samples were collected by centrifugation (8,000 × g for 10 min), washed and pooled using 0.85% saline solution. Collected pooled samples represented a composite of 30 individual chicken carcasses for each of the methods performed (modified HB and NRJ) by direct plating. Pooled samples were stored at 2°C–8°C prior to bacterial DNA extraction, which was performed within 24 h.

Furthermore, an enrichment plating procedure following United States Department of Agriculture (USDA) established guidelines for microbiological testing of poultry rinsate (United States Department of Agriculture Food Safety and Inspection Services, 2014) was performed using double strength (2X) HB and double strength (2X) NRJ broth. Enriched samples were incubated aerobically at 30°C for 48 h. After incubation, HB and NRJ selective enrichment samples were T-streaked onto modified HB and NRJ agar plates using a sterile 10-μl inoculation loop (Katz, 2012). Plates were incubated and bacterial lawns were recovered as described for direct enumeration procedures. Aliquots of the bacterial lawn were aseptically collected, washed, and pooled. Collected pooled samples represented a composite of 30 individual chicken carcasses for each of the methods performed (modified HB and NRJ) by enrichment plating. Pooled samples were stored at 2°C–8°C and bacterial DNA was extracted within 24 h.

### DNA Extraction, 16S rRNA Gene Amplicon Sequencing, and Microbiota Composition Analysis

Microbial community analysis was performed on the pooled chicken rinsate samples and the bacterial lawns recovered from both the modified HB and NRJ methods using direct enumeration and enrichment plating procedures. Metagenomic DNA was extracted from a portion of each pooled rinsate sample using the GeneJET Genomic DNA Purification Kit (Thermo Fischer Scientific, United States) according to the manufacturer’s instructions. DNA samples were quantitated using the AccuGreen High Sensitivity (HS) dsDNA Quantitation kit (Biotium, Fremont, CA, United States) with a Qubit 2.0 fluorometer (Invitrogen, Carlsbad, CA, United States). DNA sample purity was assessed from their absorbance ratios using a microvolume spectrophotometer (Denovix, Wilmington, DE, United States). DNA molecular weights were estimated by electrophoresis on agarose gels (0.8%) dyed with GelGreen Nucleic Acid Gel Stain (Biotium) and visualized on the ChemiDoc touch imaging system (BioRad, Wilmington, DE, United States). Multiplexed 16S amplicon DNA libraries were prepared with the 16S Barcoding Kit (SQK-RAB204) and sequenced with R9.4.1 flow cells (FLO-MIN106D) on a MinION instrument following instructions from the manufacturer (Oxford Nanopore, Oxford, United Kingdom). Base calling and demultiplexing was performed post-sequencing with Guppy 5.0.11 using the dna_r9.4.1_450bps_hac high accuracy model (Oxford Nanopore, Oxford, United Kingdom). Reconstruction of the microbiota composition in the Oxford Nanopore 16S rRNA gene datasets at the genus-level were performed by the UIC Research Informatics Core (Chicago, IL, United States) based on the percentage of 16S rRNA gene sequences identified in the sample. Briefly, Nanopore adaptors were removed using the Porechop v0.2.3 pipeline^1^ with a minimum trimming length of 1,000 base pairs. Sequence datasets were taxonomically annotated using the local blastn search v2.12.0 (Altschul et al., 1990; Benson et al., 2013) against the NCBI 16S ribosomal RNA sequence database.^2^ A summary of the 16S multiplex sequencing run and sample descriptions are detailed in Supplementary Table S1. The taxonomic annotation was performed using the top five alignments for each read with a minimum percent identity of 90% and an E-value < 10^−4^. Taxonomic assignment for each read was reported using the majority consensus of the top five aligned references with a required 90% query coverage for genus-level assignment. Data is presented by averaging the number of identified operational taxonomic units (OTUs) per successful replicate sequencing run. Graphical representation of the results was performed using OriginPro software (Origin-Lab, Northampton, MA). Further verification of the bacterial isolates recovered from the pooled samples after direct and enrichment procedures was confirmed by biochemical assays and microscopy. These test included the oxidase test, catalase test, Gram-stain and wet mount techniques (On et al., 2017).

## Results and Discussion

### Microbial Abundance of Consumer-Grade Whole Broiler Chicken

To determine the microbial population of whole broiler chicken carcasses purchased at retail, aerobic plating count was used to quantify the number of bacteria present in the sample. Supplementary Figure S1 shows the average bacterial counts (CFU/ml) of retail broiler chicken carcasses for each pooled chicken rinsate sample. The results show that the average (about 1.9 ×10^3^ CFU/ml) of the pooled samples (I, II, and III) is below the level of bacterial counts, ranging from 3.0 ×10^4^ to 3.3 ×10^4^ CFU/ml, as reported by the National Microbiological Baseline Data Collection Program: Young Chicken Survey (United States Department of Agriculture Food Safety and Inspection Services, 2008), properly representing consumer-grade poultry products. Although the average microbial count of several individual whole broiler chicken carcasses resulted in values above the normal level (Supplementary Figure S1), these samples represented the microbial contamination level of poultry commonly purchased at retail, within the expiration date of the product (Marmion et al., 2021).

### Microbiota Identification Using 16S rRNA Gene Amplicon Sequencing

In this study, the NRJ method performance was compared to the HB method, to validate the NRJ detection system for poultry sample testing. The modified HB method’s reported sensitivity (70.7%) is significantly higher compared to other culture-based methods, which includes the Houf method (41.5%) and Atabay and Corry method (43.9%; Merga et al., 2011). Other studies have reported that a filtration step using cellulose acetate membrane filters onto blood agar significantly increased sensitivity. However, these methods are cumbersome, costly, and show reduced specificity (Scullion et al., 2004; Hamill et al., 2008; Shah et al., 2011; Fallas-Padilla et al., 2014). Moreover, it is difficult and expensive to maintain a steady-supply of blood serum for media preparation and blood agars are frequently contaminated by swarming bacteria (Collins et al., 1996; Scullion et al., 2004). Therefore, the methods described by Collins et al. (1996), DeBoer et al. (1996), and Johnson and Murano (1999) were not evaluated since they required sub-culturing onto blood agar to carry out consistent biochemical confirmations (Ohlendorf and Murano, 2002). The specificity for membrane filtration onto blood agar is also lower compared with the modified HB (63.9%; Merga et al., 2011) when used in conjunction with the deBoer method (63.1%) and Atabay and Corry method (34.7%–39.4%; Shah et al., 2011). These findings indicate that the modified HB method showed the highest sensitivity and specificity compared to other evaluated protocols and should be used as reference for method comparison evaluations.

**Figure 1:**
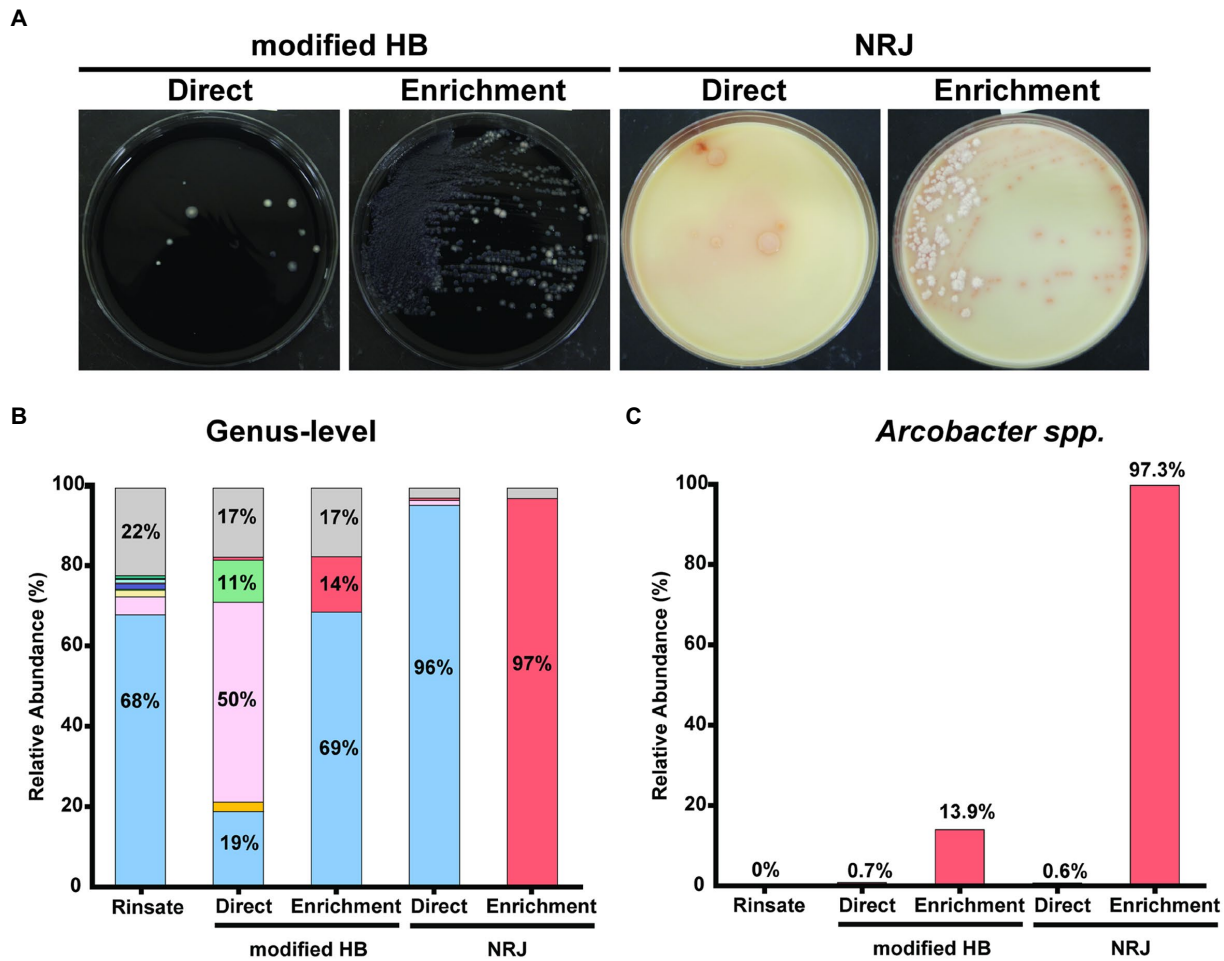
**(A)** Bacterial lawn isolated in modified HB and NRJ plating media after direct and enrichment plating procedures. All colonies using modified HB appear as 1–2 mm in diameter, white-gray, convex colonies contrasting on the black charcoal agar background for both plating procedures. Direct plating on NRJ recovered 2–3 mm, beige, circular colonies with a pinkish ring. Enrichment plating on NRJ recovered two distinct morphological colonies: 2–3 mm in diameter, white, filamentous colonies and 1–2 mm in diameter, salmon-colored colonies. **(B)** Relative abundance of predominant (>1.0%) bacterial genera with 90% query coverage found in pooled chicken rinsate samples (*n* = 30) and corresponding isolated bacterial lawns recovered from modified HB and NRJ using direct and enrichment plating methods. Represented genera are color coded: *Pseudomonas* (blue), *Carnobacterium* (pink), *Yersinia* (yellow), *Lactococcus* (green), *Aeromonas* (indigo), *Acinetobacter* (salmon), *Shewanella* (light blue), *Brochothrix* (brown), *Psychrobacter* (purple), *Vagococcus* (turquoise), *Arcobacter* (red), and other taxa (gray). Percentages are shown as text inside bars. **(C)** Relative abundance of *Arcobacter* spp. from samples described in **(B)**.

To evaluate the bacterial composition of chicken rinsates and isolates recovered using the two detection systems, genus-level identification was performed using 16S rRNA gene amplicon sequencing. Figure 1B shows the composition of chicken rinsate averaged for the three pooled samples and representing 30 individual carcasses (Figure 1B). *Pseudomonas* is the predominant microbial component, representing 68% of the taxa present. Other genera identified were *Carnobacterium* (5%), *Yersinia* (2%), and *Aeromonas* (1%). The abundance of *Arcobacter* accounted for ~0.03% of the microbial composition of the chicken rinsate (Figure 1B), we proceeded to evaluate the performance of the modified HB and NRJ *Arcobacter* detection systems, quantifying the bacterial composition of chicken rinsate samples directly plated onto the selective plating media (Figure 1A). The *Arcobacter* genus relative abundance accounted for 0.7% and 0.6% in isolates recovered on HB and NRJ plates, respectively (Figure 1C). Therefore, direct plating onto selective agar media was unable to recover *Arcobacter* likely due to the low concentrations in the sample. An enrichment procedure was employed to increase the number of *Arcobacter* bacteria by incubating chicken rinsate samples in HB and NRJ broth prior to plating on selective media. This is a common method to increase bacterial populations to detectable levels and to eliminate contaminant growth. Figures 1B,C show the relative abundance of the predominant bacterial genera (with a minimum 16S rRNA gene percent identity of 90% and a 90% query coverage) recovered from selective plating media streaked from enriched rinsate samples. Although the relative abundance of *Arcobacter* genus increased to 14% using the modified HB method, *Pseudomonas* spp. was the predominant taxa recovered (69%), demonstrating that modified HB method is not specific for *Arcobacter*. This is in accordance with previous studies showing that *Pseudomonas* spp. is the prevalent contaminant among isolates recovered from fresh meats and poultry using various culture-based methods (Merga et al., 2011; Shah et al., 2011). In the NRJ method, *Arcobacter* spp. accounted for >97% of the relative microbial abundance, which makes it a suitable *Arcobacter*-specific detection system. Microscopy and biochemical confirmation was performed to verify presumptive *Arcobacter* colonies recovered from the pooled samples (On et al., 2017). Presumptive *Arcobacter* colonies appeared as 0.5–1.0 mm in diameter, translucent gray–white, round colonies using the modified Houf method and 0.5–1.5 mm in diameter, salmon, round, convex-flat colonies using NRJ. Typical *Arcobacter* spp. appeared as Gram-negative, spiral-comma shaped bacteria that displayed darting motility under microscope were positive for the oxidase and catalase test (not shown). Our group has shown that NRJ has 97.8% inclusivity and 100.0% exclusivity when evaluating growth of select organisms associated with foods (Nguyen et al., 2020). Although, NRJ’s specificity and sensitivity has yet to be determined, we can calculate that sensitivity is at least 9 CFU/ml, based on *Arcobacter* spp. abundance in poultry (0.03%) and the number of colonies found in APC (3 ×10^4^ CFU/ml). The specificity for NRJ reported here is at least 97%, which make it the best detection system reported thus far.

A limitation in the validation of the NRJ detection system’s efficacy is the use of a minimum 90% 16S rRNA gene percent identity and 90% query coverage for taxonomic assignment at the genus-level. The traditional >95% 16S rRNA gene identity cutoff for genus-level assignment (Johnson et al., 2019) was not used in the presented work since the data was obtained using Oxford Nanopore Platform, which is prone to sequencing error (Delahaye and Nicolas, 2021). To account for this limitation, the taxonomic assignment for each read was based on the consensus of the top five references that matched within a 90% sequence identity. The relaxed threshold accommodated a greater number of sequence errors while the consensus allowed for taxon identification based on multiple references from the same taxon. Furthermore, the number of 16S rRNA gene copies is not equally represented between different taxa (Větrovský and Baldrian, 2013). Although, the variability of the 16S rRNA gene among bacterial genomes can led to bacterial composition bias, 16S amplicon sequencing was used to validate the NRJ detection system to provide an estimate of the relative abundance for the bacterial lawn recovered on the selective plating media. Compared to traditional molecular identification techniques, this approached reduced the inherent technician bias when selecting typical colonies for further confirmation. Regardless, future validation studies of the NRJ detection system should explore molecular characterization of the bacterial colonies to identify *Arcobacter* at the species-level.

These results show that the NRJ detection system is an effective method able to recover *Arcobacter* colonies with little to no interference from contaminants in complex samples, such as retail chicken. Furthermore, *Arcobacter* spp. was detected in the normal microbiota of consumer-grade poultry carcasses, which suggests these pathogens are relevant to human health and food safety. The NRJ method is reliable and should be considered the new gold standard for *Arcobacter-*specific culture-based method with applications in food sampling, specifically in the case of poultry. Further testing is needed to determine the NRJ detection system’s potential suitability beyond food-related applications, such as clinical and environmental sampling. Moreover, inter-laboratory comparisons for method-performance should be conducted evaluating different sample matrices associated with this pathogen. The NRJ detection system can be used as the standard culture media for *Arcobacter* isolation, as this emerging food pathogen trend continues to grow, which requires further evaluation by the United States Food and Drug Administration and United States Department of Agriculture, along with the International Organization for Standardization.

## Data Availability Statement

The raw data supporting the conclusions of this article will be made available by the authors, without undue reservation.

## Author Contributions

OJ, LR, and PN were responsible for conceiving the initial design of the study and drafted the initial manuscript. PN contributed to the experimental implementation, data analysis, and conception of the paper. KT was involved in data acquisition. Bioinformatics analysis in the project described was performed by the UIC Research Informatics Core. All authors contributed to the article, revised the manuscript critically, and approved the submitted version.

## Funding

This research was supported by the National Institutes of Health R15GM131292 and R01AI151152 to OJ. The bioinformatics analysis in the project described was performed by the UIC Research Informatics Core, supported in part by NCATS through Grant UL1TR002003. R & F Products, Inc. suppled the NRJ media used in the project and they have expressed their willingness to supply their product for external researchers to evaluate the performance of NRJ for other sampling applications.

## Conflict of Interest

PN and LR were employed by R & F Products, Inc.

The remaining authors declare that the research was conducted in the absence of any commercial or financial relationships that could be construed as a potential conflict of interest.

## Publisher’s Note

All claims expressed in this article are solely those of the authors and do not necessarily represent those of their affiliated organizations, or those of the publisher, the editors and the reviewers. Any product that may be evaluated in this article, or claim that may be made by its manufacturer, is not guaranteed or endorsed by the publisher.

## Footnotes

^1^ https://github.com/rrwick/Porechop

^2^ https://ftp.ncbi.nlm.nih.gov/blast/db/README
