# Supplementary material for: NRJ Media as the Gold-Standard Arcobacter-Specific Detection System: Applications in Poultry Testing
Source: Front Microbiol. 2022 Jun 21;13:903079. doi: 10.3389/fmicb.2022.903079 (PMC9253625; doi:10.3389/fmicb.2022.903079)
Supplement: Supplementary file 1 [file Table_1.DOCX]

Supplementary Material

| **Table S1**. Quantitative and qualitative summary of 16S sequencing run. | | |
| --- | --- | --- |
| **Barcode** | **Description** | **Direct/ Enrichment (D/E)** |
| 16S01 | Rinsate 1 (n = 10) | N/A |
| 16S02 | Rinsate 2 (n = 10) | N/A |
| 16S03 | Rinsate 3 (n = 10) | N/A |
| 16S04 | modified Houf (n = 30) | D |
| 16S05 | modified Houf (n = 30) | D |
| 16S06 | modified Houf (n = 30) | E |
| 16S07 | modified Houf (n = 30) | E |
| 16S08 | NRJ (n = 30) | D |
| 16S10 | NRJ (n = 30) | D |
| 16S09 | NRJ (n = 30) | E |
| 16S11 | NRJ (n = 30) | E |
| 16S rRNA gene amplicon sequence runs for composited/replicate samples are indicated as follows: (i) Rinsate 1, Rinsate 2, and Rinsate 3; (ii) 16S04 and 16S05, (iii) 16S06 and 16S07, (iv) 16S08 and 16S10, (v) 16S09 and 16S11. | | |


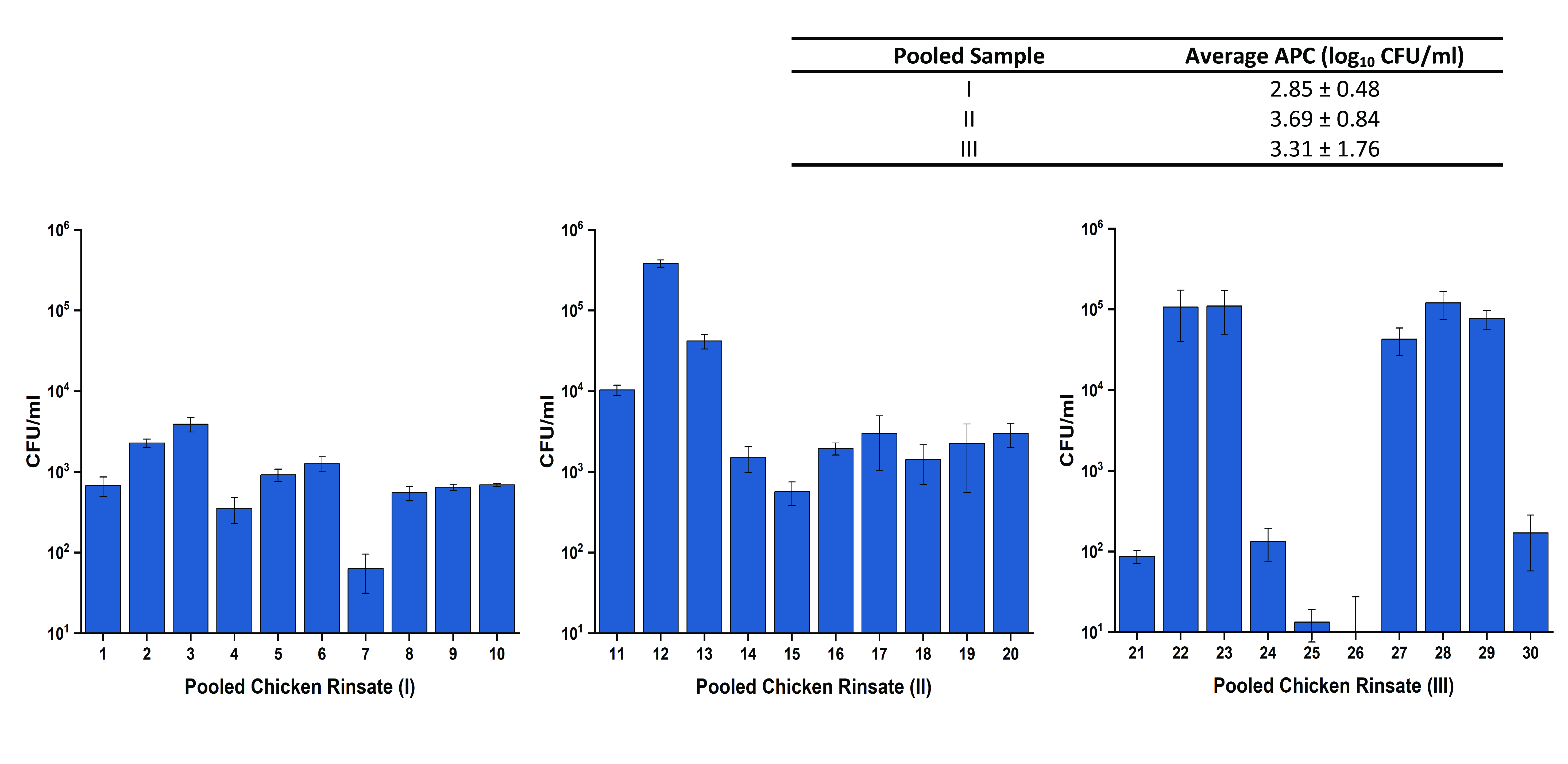


**Supplementary Figure 1.** Aerobic plate count (CFU/ml) of individual whole broiler chicken carcasses purchased at retail. Pooled chicken rinsate samples (I-III) represent a composite of 10 samples.
